# Psychological Investigations of Humor and Laughter: Honoring the Research Contributions of Professor Rod Martin: Opening Comments

**DOI:** 10.5964/ejop.v12i3.1213

**Published:** 2016-08-19

**Authors:** Nicholas A. Kuiper

**Affiliations:** a Department of Psychology, University of Western Ontario, London, Ontario, Canada

Welcome to this special issue of *Europe’s Journal of Psychology* (EJOP), which honors the many psychological research contributions on humor and laughter made by Professor Rod Martin. After completing his graduate training at the University of Waterloo in 1984, Dr. Martin became a faculty member in the clinical psychology program at the University of Western Ontario. Professor Martin then remained at Western for many years, conducting research on humor and laughter, until his very recent retirement in the summer of 2016.

In a distinguished research career which spans over 30 years, Professor Martin has published a large number of high impact research articles, chapters, and scholarly books on humor and laughter. Representative illustrations of these contributions are shown in Table 1. This table is organized accordingly to the various topic areas within the domains of humor and laughter research that Professor Martin has explored since the early to mid-1980s.

**Table 1:** Humor and Laughter Topics Investigated by Professor Rod Martin

Books on Humor and Laughter Lefcourt, H. M., & Martin, R. A. (1986). *Humor and life stress: Antidote to adversity*. New York, NY, USA: Springer. Martin, R. A. (2007). *The psychology of humor: An integrative approach*. Burlington, MA, USA: Elsevier Academic Press. [Translated into Spanish, Korean, Japanese and Russian] Assessment of Humor and Laughter Martin, R. A. (1996). The Situational Humor Response Questionnaire (SHRQ) and Coping Humor Scale (CHS): A decade of research findings. *Humor: International Journal of Humor Research, 9*, 251-272. Martin, R. A., Puhlik-Doris, P., Larsen, G., Gray, J., & Weir, K. (2003). Individual differences in uses of humor and their relation to psychological well-being: Development of the Humor Styles Questionnaire. *Journal of Research in Personality, 37*, 48-75. Edwards, K. R., & Martin, R. A. (2014). The conceptualization, measurement, and role of humor as a character strength in positive psychology. *Europe’s Journal of Psychology, 10*, 505-519. Humor and Physical Health Martin, R. A. (2001). Humor, laughter, and physical health: Methodological issues and research findings. *Psychological Bulletin, 127*, 504-519.10.1037/0033-2909.127.4.50411439709 Martin, R. A. (2002). Is laughter the best medicine? Humor, laughter, and physical health. *Current Directions in Psychological Science*, *11*, 216-220. [invited article] Martin, R. A. (2004). Sense of humor and physical health: Theoretical issues, recent findings, and future directions. *Humor: International Journal of Humor Research, 17*, 1-19. Edwards, K. R., & Martin, R. A. (2012). Do humorous people take poorer care of their health? Associations between humor styles and substance use. *Europe’s Journal of Psychology, 8*, 1-12. Humor, Psychological Well-Being and Psychopathology Martin, R. A., & Lefcourt, H. M. (1983). Sense of humor as a moderator of the relation between stressors and moods. *Journal of Personality and Social Psychology, 45*, 1313-1324. Kuiper, N. A., Martin, R. A., & Olinger, L. J. (1993). Coping humor, stress, and cognitive appraisals. *Canadian Journal of Behavioral Science, 25*, 81-96. Kuiper, N. A., Martin, R. A., Olinger, L. J., Kazarian, S. S., & Jette, J. L. (1998). Sense of humor, self-concept, and psychological well-being in psychiatric inpatients. *Humor: International Journal of Humor Research, 11*, 357-381. Frewen, P. A., Brinker, J., Martin, R. A., & Dozois, D. J. A. (2008). Humor styles and personality-vulnerability to depression. *Humor: International Journal of Humor Research, 21,* 179-195. Dozois, D. J. A., Martin, R. A., & Bieling, P. J. (2009). Early maladaptive schemas and adaptive/maladaptive styles of humor. *Cognitive Therapy and Research, 33*, 585-596. Dozois, D. J. A., Martin, R. A., & Faulkner, B. (2013). Early maladaptive schemas, styles of humor, and aggression. *Humor: International Journal of Humor Research, 26*(1), 97-116. Humor, Positive Psychology and Quality of Life Kuiper, N. A., Martin, R. A., & Dance, K. A. (1992). Sense of humor and enhanced quality of life. *Personality and Individual Differences, 13*, 1273-1283. Kuiper, N. A., & Martin, R. A. (1998). Is sense of humor a positive personality characteristic? In W. Ruch (Ed.), *The sense of humor: Explorations of a personality characteristic* (Humor Research Series) (pp. 159-178). Berlin, Germany: Mouton de Gruyter. Kuiper, N. A., & Martin, R. A. (1998). Laughter and stress in daily life: Relation to positive and negative affect. *Motivation and emotion* [Special issue on affect and self-regulation], *22*, 133-153. Edwards, K. R., & Martin, R. A. (2010). Humor creation ability and mental health: Are funny people more psychologically healthy? *Europe’s Journal of Psychology, 3*, 196-212. Veselka, L., Schermer, J. A., Martin, R. A., & Vernon, P. A. (2010). Laughter and resiliency: A behavioral genetic study of humor styles and mental toughness. *Twin Research and Human Genetics, 13*, 442-449.10.1375/twin.13.5.44220874465 Humor, Personality and other Individual Difference Attributes Martin, R. A., & Kuiper, N. A. (1999). Daily occurrence of laughter: Relationships with age, gender, and Type A personality. *Humor: International Journal of Humor Research, 12*, 355-384. Martin, R. A., Lastuk, J. M., Jeffery, J., Vernon, P. A., & Veselka, L. (2012). Relationships between the dark triad and humor styles: A replication and extension. *Personality and Individual Differences, 52,* 178-182. Schermer, J. A., Martin, R. A., Martin, N. G., Lynskey, M., & Vernon, P. A. (2013). The general factor of personality and humor styles. *Personality and Individual Differences, 54*(8), 890-893. Martin, R. A. (2014). Humor and gender: An overview of psychological research. In D. Chiaro & R. Baccolini (Eds.), *Gender and humor: Interdisciplinary and international perspectives* (pp. 123-146). New York, NY, USA: Routledge. Humor and Developmental Issues Martin, R. A. (1989). Humor and the mastery of living: Using humor to cope with the daily stresses of growing up. In P. E. McGhee (Ed.), *Humor and children's development: A practical guide to applications*. New York, NY, USA: Haworth Press, 135-154. Kazarian, S. S., Moghnie, L., & Martin, R. A. (2010). Perceived parental warmth and rejection in childhood as predictors of humor styles and subjective happiness. *Europe’s Journal of Psychology, 3*, 71-93. Humor and Cross-Cultural Issues Kazarian, S. S., & Martin, R. A. (2006). Humor styles, culture-related personality, well-being, and family adjustment among Armenians in Lebanon. *Humor: International Journal of Humor Research, 19*, 405-423. Chen, G. H., & Martin, R. A. (2007). A comparison of humor styles, coping humor, and mental health between Chinese and Canadian university students. *Humor: International Journal of Humor Research, 20*, 215-234. Chen, G., Watkins, D., & Martin, R. A. (2013). Sense of humor in China: The role of individualism, collectivism, and facework. *Psychologia, 56,* 57-70. Humor and Behavioral Genetics Bressler, E. R., Martin, R. A., & Balshine, S. (2006). Production and appreciation of humor as sexually selected traits. *Evolution and Human Behavior, 27*, 121-130. Vernon, P. A., Martin, R. A., Schermer, J. A., & Mackie, A. (2008). A behavioral genetic investigation of humor styles and their correlations with the Big-5 personality dimensions. *Personality and Individual Differences, 44*, 1116-1125. Veselka, L., Schermer, J. A., Martin, R. A., Cherkas, L. F., Spector, T. D., & Vernon, P. A. (2010). A behavioral genetic study of relationships between humor styles and the six HEXACO personality factors. *Europe’s Journal of Psychology, 3*, 9-33. Humor in Social Interactions and Close Relationships Yip, J. A., & Martin, R. A. (2006). Sense of humor, emotional intelligence, and social competence. *Journal of Research in Personality, 40*, 1202-1208. Campbell, L., Martin, R. A., & Ward, J. R. (2008). An observational study of humor use while resolving conflict in dating couples. *Personal Relationships, 15*, 41-55. Caird, S., & Martin, R. A. (2014). Relationship-focused humor styles and relationship satisfaction in dating couples: A repeated-measures design. *Humor: International Journal of Humor Research, 27*(2), 227-247. Humor and Professional Comedians Greengross, G., Martin, R. A., & Miller, G. (2012). Personality traits, intelligence, humor styles, and humor production ability of professional stand-up comedians compared to college students. *Psychology of Aesthetics, Creativity, and the Arts, 6*, 74-82. Greengross, G., & Martin, R. A., & Miller, G. (2012). Childhood experiences of professional comedians: Peer and parent relationships and humor use. *Humor: International Journal of Humor Research, 25*, 491-505. Humor as an Intervention Technique Martin, R. A. (1996). Humor as therapeutic play: Stress-moderating effects of humor. *Journal of Leisurability, 23*, 8-15. Rudnick, A., Kohn, P. M., Edwards, K. R., Podnar, D., Caird, S., & Martin, R. A. (2014). Humour-related interventions for people with mental illness: A randomized controlled pilot study. *Community Mental Health Journal, 50,* 737-742.10.1007/s10597-013-9685-424337476

What is immediately apparent, even from a cursory examination of Table 1, is the extremely broad range of humor and laughter topics that Professor Martin has investigated. Over the years, his theoretical and research contributions have been at the forefront of psychologically-based humor investigations, resulting in considerable advances in our understanding of many different psychological aspects of humor and laughter. For example, in addition to writing a definitive book in 2007 that provided a broad overview of theoretical approaches and research on the psychology of humor and laughter, Dr. Rod Martin has also developed several assessment measures for humor that have become the leading standards in the field, such as the Humor Styles Questionnaire.

Professor Martin has also made substantial theoretical and research contributions to several other longstanding issues in the humor and laughter research domain, including clarifications and elaborations regarding the various relationships between humor and physical health, humor and psychological well-being, and humor and psychopathology. Further themes that have been prominent in Professor Martin‘s research program have integrated his work on humor with a broader positive psychology perspective. Dr. Martin and his colleagues have also mapped out how various personality characteristics and other individual difference attributes may relate to sense of humor, particularly as expressed via the four humor styles. Some of this work has explored developmental issues in humor, whereas other studies have focused on the degree to which sense of humor may display cross-cultural variations.

Further aspects of Professor Martin’s work has addressed the nature versus nurture distinctions by examining humor styles from a behavioral genetics perspective, as well as considering how professional comedians may differ from non-comedians in their upbringing, the expression of certain personality traits and their humor production ability. Dr. Martin has also advanced our understanding of how humor and laughter may be involved in social interactions and close relationships, as well as the use of humor as a possible intervention technique.

Overall, this very brief synopsis of Professor Martin’s many research activities and accomplishments over the past 30 or so years clearly reveals both the breadth and depth of his contributions to our psychological understanding of humor and laugher. In turn, his contributions now provide a robust theoretical-empirical framework that can be built upon by future investigators, as they continue to explore psychological aspects of humor and laughter.

Professor Martin’s legacy in the humor research domain is very strong, and this special issue honors his contributions by presenting an in-depth interview with Dr. Martin (Martin & Kuiper, 2016, this issue). In this interview, Professor Martin discusses several of his main humor research contributions over the past 30 years, and provides both a personal and historical perspective on his career highlights and accomplishments. In addition, Dr. Martin also provides many insightful comments regarding some of the most significant issues facing contemporary humor and laughter researchers today.

## Articles in the Current Special Issue

In further recognition of Dr. Martin’s many research achievements, this special humor issue of *Europe’s Journal of Psychology* offers a set of contemporary psychological studies investigating various aspects of humor and laughter. Table 2 provides a brief summary of several key characteristics for each of the papers in this special issue. These characteristics include the humor constructs being investigated in that study, the sample employed, and the main issues under consideration. As can be seen in Table 2, the majority of the studies in this special issue (see the first 9 listed in this table) build upon Dr. Martin’s prominent humor styles model. In turn, the last 3 papers listed in Table 2 provide samples of additional contemporary work in the humor domain that does not derive directly from Professor Martin’s work, but rather focuses on laughter research, therapeutic medical clowning, and the semiotic analysis of jokes.

**Table 2:** Overview of Papers in the Special Humor Issue (EJOP August 2016)

Investigators	Humor	Sample	Main Issues Examined
1. Ford, Lappi & Holden	Affiliative Humor Self-Enhancing Humor Aggressive Humor Self-Defeating Humor	Sample of 194 United States residents	Associations of humor styles with happiness and personality characteristics including extraversion, locus of control, optimism and self-esteem.
2. Hampes	Affiliative Humor Self-Enhancing Humor Aggressive Humor Self-Defeating Humor	112 college students in the United States	Associations of humor styles with forgiveness.
3. Rnic, Dozois & Martin	Affiliative Humor Self-Enhancing Humor Aggressive Humor Self-Defeating Humor	208 university students in Canada	Associations of humor styles with cognitive distortions and depression in social and achievement-related contexts.
4. Zeigler-Hill, McCabe & Vrabel	Affiliative Humor Self-Enhancing Humor Aggressive Humor Self-Defeating Humor	594 university students in the United States	Associations between humor styles and pathological personality traits in DSM-5, including negative affectivity, detachment, antagonism, disinhibition and psychoticism.
5. Fox, Hunter & Jones	Affiliative Humor Self-Enhancing Humor Aggressive Humor Self-Defeating Humor	1234 adolescents (aged 11-13) enrolled in secondary schools in the U.K.	Longitudinal associations between humor styles and psychosocial adjustment in adolescence.
6. DiDonato & Jakubiak	Humorous behaviors pertaining to Affiliative, Self-Enhancing, Aggressive and Self-Defeating Humor Styles	Community sample (*n* = 175) and university students (*n* = 149) in the United States	How romantic motives (short versus long term) affect use of and response to adapative and maladaptive humor.
7. Hahn & Campbell	Affiliative Humor Self-Enhancing Humor Aggressive Humor Self-Defeating Humor	116 heterosexual married couples in Canada (*n* = 232)	Degree of similarity in humor styles between spouses, and the role of self-esteem as a potential moderator of degree of similarity.
8. James & Fox	Self-Enhancing Humor Self-Defeating Humor	10 children (aged 8-11 years) at a U.K. primary school	Qualitative in-depth interviews to determine childrens’ understanding of self-focused humor styles (self-enhancing humor and self-defeating humor).
9. Ruch & Heintz	Affiliative Humor Self-Enhancing Humor Aggressive Humor Self-Defeating Humor Humorous Behavior Q-Sort Deck, Literary Comic Styles	Combined several German speaking samples for a total of 1101 participants.	Develops and reports on the psychometric properties of a German version of the Humor Styles Questionnaire. Also examined relationships between HSQ humor styles and other measures of humor (e.g., Humorous Behavior Q-sort Deck, Literary Comic Styles)
10. Svebak	Laughter	81 university students from Norway (in two studies)	Examined the consequences of laughter upon trunk compression and cortical activation.
11. Dionigi & Canestrari	Medical clowning interventions in clinical health care settings	Review of current research literature on effectiveness of medical clowning	Summarizes effects of medical clowning on adult patients, relatives and medical staff.
12. Berger	Jokes	Semiotic analyses of the joke	Typology of 45 techniques of humor in 4 categories (e.g., Logic, Identity, & Action) used to analyze a joke.

What is quite evident across all of the papers in this special issue is the extensive nature of the humor research now being conducted. As shown in Table 2, participants are drawn from many different countries (Canada, Germany, Norway Switzerland, United Kingdom, and United States), and include samples composed of children, adolescents, adults, and couples. The novel themes examined in these studies include associations of humor styles with: (1) happiness and other personality characteristics (Ford, Lappi, & Holden, 2016, this issue), (2) forgiveness (Hampes, 2016, this issue), (3) cognitive distortions and depression (Rnic, Dozois, & Martin, 2016, this issue), (4) pathological personality traits in the DSM-5 (Zeigler-Hill, McCabe, & Vrabel, 2016, this issue), (5) psychosocial adjustment across time in adolescents (Fox, Hunter, & Jones, 2016, this issue), and (6) short and long-term romantic motives (DiDonato & Jakubiak, 2016, this issue).

Other novel aspects of humor styles examined in the current set of studies include: (7) the degree of similarity in humor styles between spouses (Hahn & Campbell, 2016, this issue), (8) childrens’ understanding of self-evaluative and self-defeating humor styles (James & Fox, 2016, this issue), and (9) adaptations of the Humor Styles Questionnaire for use with German speaking populations (Ruch & Heintz, 2016, this issue).

Moving beyond a focus on the humor styles, the remaining studies in this special issue explore: (10) links between laughter, trunk compression and cortical activation (Svebak, 2016, this issue), (11) the effects of medical clowning on patients and staff (Dionigi & Canestrari, 2016, this issue), and (12) the use of a typology of 45 different techniques to analyze jokes (Berger, 2016, this issue).

More generally, as you read through these articles presented in this special humor issue of *EJOP*, I hope you find them to be enjoyable, informative and enlightening. In addition to honoring Professor Rod Martin’s research work, this collection of articles also provides an intriguing slice of contemporary theory and research on various psychological aspects of humor and laughter. As such, reading about this work may inspire you to also conduct further research on some of the topics described herein. However, in doing so, it should be remembered that the topics covered in this special issue represent only a very small portion of the exponentially increasing number of psychologically-based studies on humor and laughter published in the past few years. This additional research also warrants further detailed consideration as the field moves forward.

## Concluding Comments

While preparing this special issue, I received many positive comments from other humor investigators about the stellar quality and high impact of the many research contributions made by Professor Martin throughout his career. Notably, many of these investigators also spontaneously commented on the very gracious interpersonal style that is a hallmark of Professor Martin’s professional interactions with others. His positive demeanor, coupled with a strong ability and desire to unravel the many mysteries still surrounding our use of humor and laughter, helps make Professor Martin a truly outstanding humor scholar. His work has greatly advanced our knowledge and understanding of many different facets of humor and laughter, and we would all like to wish him the best as he begins his retirement.

I would also like to thank all of the reviewers and contributors to this special humor issue for their assistance throughout the many stages of this project. Their co-operation, patience and thoughtfulness certainly helped to make this special issue a very fitting tribute to Professor Martin’s research career and accomplishments. Finally, I would also like to offer considerable thanks to the chief editor of *EJOP*, Vlad Glăveanu, who has very capably helped me throughout this special issue project. It should be noted that this is the third in a series of special humor issues that *Europe’s Journal of Psychology* has published (see also Kuiper, 2010 and Kuiper 2014). For all three issues, the chief editor’s encouragement and unwavering support has made this editorial task immensely easier.

## References

[r1] Berger A. A. (2016). Three holy men get haircuts: The semiotic analysis of a joke. Europe’s Journal of Psychology, 12, 489-497. doi:10.5964/ejop.v12i3.1042 PMC4991053 27547262

[r2] DiDonato T. E. Jakubiak B. K. (2016). Strategically funny: Romantic motives affect humor style in relationship initiation. Europe’s Journal of Psychology*,* 12, 390-405. doi:10.5964/ejop.v12i3.1105 PMC4991047 27547256

[r3] Dionigi A. Canestrari C. (2016). Clowning in health care settings: The point of view of adults. Europe’s Journal of Psychology*,* 12, 473-488. doi:10.5964/ejop.v12i3.1107 PMC4991052 27547261

[r4] Ford T. E. Lappi S. K. Holden C. J. (2016). Personality, humor styles and happiness: Happy people have positive humor styles. Europe’s Journal of Psychology*,* 12, 320-337. doi:10.5964/ejop.v12i3.1160 PMC4991042 27547251

[r5] Fox C. L. Hunter S. C. Jones S. E. (2016). Longitudinal associations between humor styles and psychosocial adjustment in adolescence. Europe’s Journal of Psychology*,* 12, 377-389. doi:10.5964/ejop.v12i3.1065 PMC4991046 27547255

[r6] Hahn C. M. Campbell L. J. (2016). Birds of a feather laugh together: An investigation of humour style similarity in married couples. Europe’s Journal of Psychology*,* 12, 406-419. doi:10.5964/ejop.v12i3.1115 PMC4991048 27547257

[r7] Hampes W. (2016). The relationship between humor styles and forgiveness. Europe’s Journal of Psychology*,* 12, 338-347. doi:10.5964/ejop.v12i3.1012 PMC4991043 27547252

[r8] James L. A. Fox C. L. (2016). Children’s understanding of self-focused humor styles. Europe’s Journal of Psychology*,* 12, 420-433. doi:10.5964/ejop.v12i3.1067 PMC4991049 27547258

[r9] Kuiper N. A. (2010). Introductory comments: Special Issue of EJOP (August 2010) on Humor Research in Personality and Social Psychology. Europe's Journal of Psychology, 6, 1–8. doi:. 10.5964/ejop.v6i3.205

[r10] Kuiper N. A. (2014). Investigating the role of humor in psychological health and well-being: Opening comments. Europe's Journal of Psychology, 10, 408–411. 10.5964/ejop.v10i3.809

[r11] Martin R. Kuiper N. A. (2016). Three decades investigating humor and laughter: An interview with Professor Rod Martin. Europe’s Journal of Psychology*,* 12, 498-512. doi:10.5964/ejop.v12i3.1119 PMC4991054 27547263

[r12] Rnic K. Dozois D. J. A. Martin R. A. (2016). Cognitive distortions, humor styles, and depression. Europe’s Journal of Psychology*,* 12, 348-362. doi:10.5964/ejop.v12i3.1118 PMC4991044 27547253

[r13] Ruch W. Heintz S. (2016). The German version of the Humor Styles Questionnaire: Psychometric properties and overlap with other styles of humor. Europe’s Journal of Psychology*,* 12, 434-455. doi:10.5964/ejop.v12i3.1116 PMC4991050 27547259

[r14] Svebak S. (2016). Consequences of laughter upon trunk compression and cortical activation: Linear and polynomial relations. Europe’s Journal of Psychology*,* 12, 456-472. doi:10.5964/ejop.v12i3.1102 PMC4991051 27547260

[r15] Zeigler-Hill V. McCabe G. A. Vrabel J. K. (2016). The dark side of humor: DSM-5 pathological personality traits and humor styles. Europe’s Journal of Psychology*,* 12, 363-376. doi:10.5964/ejop.v12i3.1109 PMC4991045 27547254

